# Saccadic latency in Parkinson's disease correlates with executive function and brain atrophy, but not motor severity

**DOI:** 10.1016/j.nbd.2011.01.032

**Published:** 2011-07

**Authors:** Robert Perneczky, Boyd C.P. Ghosh, Laura Hughes, Roger H.S. Carpenter, Roger A. Barker, James B. Rowe

**Affiliations:** a Department of Psychiatry and Psychotherapy, Technische Universität München, Munich, Germany; b Department of Clinical Neurosciences, University of Cambridge, Cambridge, UK; c Department of Physiology, Development and Neuroscience, University of Cambridge, Cambridge, UK; d Cambridge Centre for Brain Repair, Cambridge, UK; e Medical Research Council Cognition and Brain Sciences Unit, Cambridge, UK; f Behavioural and Clinical Neuroscience Institute, University of Cambridge, Cambridge, UK

**Keywords:** Parkinson's disease, Saccade, LATER model, Prefrontal cortex, Executive function, Voxel-based morphometry

## Abstract

Brain regions related to saccadic control are affected by Parkinson's disease (PD) pathology and a relationship between abnormal saccades and cognitive features of PD has been suggested. We measured the latency of visually-evoked saccades, and correlated best-fit parameters in a LATER neuronal decision model μ and σ (mean and SD of the distribution of reciprocal latency, i.e. speed of response), and σ_E_ (SD of the early component) with motor function, cognition and grey matter volume in 18 patients with PD and 17 controls. There was a negative correlation between verbal fluency and σ; no correlation was found between motor function and any of the latency parameters. Higher μ (shorter latency) positively correlated with grey matter volume in the prefrontal cortex, the cerebellar vermis, and the fusiform gyrus. There was a negative correlation between σ and grey matter volume in the frontal and parietal eye fields, the premotor cortex, and the lateral prefrontal cortex. σ_E_ negatively correlated with grey matter volume in the frontal eye fields and the middle frontal gyrus. Our behavioural and imaging findings point to an association between saccade latency, executive function and the structural integrity within a well-defined oculomotor network.

## Research highlights

► Saccadic latency is related to brain regions affected by Parkinson's disease. ► Information on the underlying neural decision process can be derived from saccades. ► We present the grey matter volume and cognitive correlates of saccade alterations. ► Implications for the neural decision process are discussed.

## Introduction

Although Parkinson's disease (PD) is clinically characterised by tremor, rigidity, and bradykinesia, PD also affects oculomotor control including abnormal saccades (Jankovic, 2008). PD pathology affects both cortical (Pierrot-Deseilligny et al., 2003) and subcortical (Hikosaka et al., 2000) brain regions associated with saccade control, and therefore saccadic alterations have gained great interest as a potential biomarker in PD and related disorders (Ali et al., 2006; Antoniades et al., 2007; Michell et al., 2006; Temel, 2008; Temel et al., 2009). Furthermore, there is evidence for the association between abnormal saccades and non-motor features of PD such as cognitive impairment and mood disorder (Mosimann et al., 2005).

Visually-evoked saccades are easily accessible to clinical observation or laboratory measurement, with well delineated dynamic properties and high reproducibility (Roy-Byrne et al., 1995). Measurement of the interval between the presentation of a target stimulus and the onset of a saccade (i.e. the latency) has in particular been studied to understand the underlying pathophysiology. Latencies are far longer than would be predicted by synaptic delays and nerve conduction, and appear to be dominated by the time needed by higher cortical levels to make the decision that initiates a saccade (Carpenter, 1981). In this respect the basal ganglia are also important as they have been shown to permit saccade initiation by removing tonic inhibition of the superior colliculus (reviewed in Leigh and Kennard, 2004). Thus, both cortical and sub-cortical pathology may contribute to the saccadic abnormalities associated with PD.

Several studies suggest that saccadic latency is abnormal in a range of neurodegenerative disorders including PD (Ali et al., 2006; Antoniades et al., 2007; Michell et al., 2006; Rivaud-Pechoux et al., 2000). Moreover, significant effects of dopaminergic drugs (Michell et al., 2006) and subthalamic nucleus stimulation (Temel et al., 2009) on saccadic latency have also been reported, although these effects of dopaminergic treatment on saccade latency are not consistent (Rascol et al., 1989).

Latencies, like other reaction times, also vary randomly from trial to trial and this variability is in itself informative about the saccadic decision process. The reciprocal of the latency (the speed of response) follows a Gaussian distribution, consistent with the LATER model of neural decision making (Carpenter and Williams, 1995). Under this model, a neural decision signal rises at a rate *r* in response to a stimulus until it reaches a threshold, at which point the response is initiated. It is found that the rate *r* varies randomly from trial to trial, with a Gaussian distribution (mean μ, variance σ), generating the skewed distribution of latency that is characteristic of all reaction times. This means that the cumulative frequency lies on a straight line when plotted on a probability scale as a function of reciprocal latency (figure 1 in Carpenter and McDonald, 2007 and Supplement 1). A small sub-population of saccades can often also be observed, with a shorter latency, particularly under circumstances such as urgency or impaired cognition. These early saccades are generated from a distinct but similar parallel decision process, with mean rate of rise of zero, and variance σ_E_, and can be seen on a reciprobit plot as a shallower line intersecting the main slope.

Although changes in saccades are described in neurodegenerative disorders, we lack essential information about the cognitive and anatomical correlates of these changes. Therefore, in the present study we aimed to (1) define the neuroanatomical substrate for the saccadic latency changes in PD; (2) test the hypothesis that alterations of saccadic latency correlate with specific cognitive and motor changes in PD; and (3) replicate the reported latency differences between patients with PD and elderly control subjects and the effects of dopaminergic treatment on saccadic latency.

## Materials and methods

### Subjects

Eighteen patients with idiopathic PD according to UK brain bank clinical diagnostic criteria and 17 elderly healthy controls without any other neurological or psychiatric illness including dementia were recruited at the Cambridge Centre for Brain Repair. All participants were subjected to a standardised clinical assessment protocol. Participants were assessed on two sessions; they were asked to take their usual medication on one day to be in a dopaminergic ‘on’ state. On the other day they were asked to stop their medication and were therefore in a relative ‘off’ state. Controls were also scanned twice and randomly assigned to a nominal ‘on’ or ‘off’ session. The neuropsychological assessment after the ‘on’ scanning session included the Mini-Mental-State Examination (MMSE) and tests of animal fluency (animals/1 min) and letter fluency (words beginning with the letter p/1 min). The severity of PD motor symptoms was assessed by the Unified PD rating scale (UPDRS) motor subscale III immediately before scanning (full details in Supplement 1).

### Data acquisition, pre-processing and analysis

Saccadometry was conducted according to a published standard protocol (Michell et al., 2006). We used a head-mounted saccadometer device using binocular infra-red reflection from the limbus of each eye to record participants' visually evoked saccades in response to the horizontal displacement of a red laser point target. Latency distribution analyses including the estimation of the best-fit LATER parameters were carried out in SPIC software. Structural MRI images were obtained on a Siemens 3T scanner; image pre-processing was performed in SPM5 software.

Voxel-based morphometry (VBM) (Ashburner and Friston, 2000) in SPM5 software used general linear models to estimate the associations between the three LATER parameters and grey matter volume in patients and controls. A family-wise error rate correction was applied in order to control for false-positive findings due to multiple comparisons. Only voxels with a corrected *p* < 0.05 were regarded as significant. Both positive and negative correlations were considered. Additionally, each analysis was repeated subsequently with an implicit mask for the contrast of “controls vs. patients”, in order to determine whether significant findings lay within brain regions with grey matter reduction in patients versus controls. A voxel might be atrophic by virtue of PD, or correlation with saccade/cognition, or both. The implicit masking procedure allows one to determine whether an area with correlation with a saccade parameter is an area that is also atrophic overall in the PD group. It does not adjust the statistical values of identified voxels (it is not a region-of-interest analysis and does not use small volume correction) but is akin to a conjunction analysis. In other words, the voxel grey matter correlates with mean corrected saccade parameter with family-wise error rate correction and at the same time is trended overall to be atrophic in PD.

Mean values of the LATER parameters were analysed for significant differences between the two groups and between patients ‘on’ and ‘off’ l-dopa. Significant associations between the LATER parameters μ, σ, and σ_E_ and the variables UPDRS III (‘on’ and ‘off’), MMSE, animal and letter fluency, and whole brain volume were also tested (full details in Supplement 1).

## Results

### Behaviour and saccadometry

Study sample characteristics are presented in Table 1. There were no significant differences in age or gender distribution between the groups. Patients and controls scored similarly on cognitive tests including the MMSE, and the animal and letter fluency tests. Grey matter volumes did not differ significantly between patients and controls although the absolute mean value was 112 ml less in patients.

**Fig. 1:**
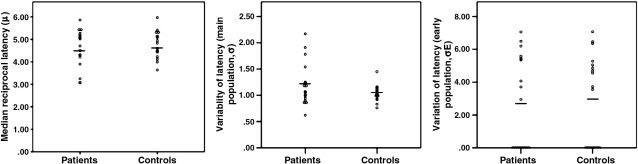
Scatter plots of the three LATER parameters in the patient and control groups (‘on’ state). Solid lines indicate mean values.

Regarding the median reciprocal latency (speed of response, μ) and variability of the main saccade population (σ) or early saccade population (σ_E_) latency parameters from the LATER model, there were no significant differences between the control group and ‘on’ patients, between ‘on’ and ‘off’ patients, or between controls and ‘off’ patients (Table 2, Fig. 1).

In patients, there was no significant correlation between any of the three saccade parameters and the degree of motor dysfunction as measured by the UPDRS III in the ‘on’ (μ: *r* = −0.12, *p* = 0.33; σ: *r* = 0.05, *p* = 0.42; σ_Ε_: *r* = −0.23, *p* = 0.19; *N* = 18) or ‘off’ conditions (μ: *r* = 0.02, *p* = 0.47; σ: *r* = −0.08, *p* = 0.39; σ_Ε_: *r* = 0.08, *p* = 0.38; *N* = 18). Furthermore, there was no significant correlation between whole brain volume and any of the three parameters in patients in the ‘on’ and the ‘off’ conditions (‘on’ condition, μ: *r* = −0.15, *p* = 0.31; σ: *r* = 0.23, *p* = 0.21; σ_Ε_: *r* = 0.10, *p* = 0.37; *N* = 18; ‘off’ condition, μ: *r* = 0.03, *p* = 0.46; σ: *r* = 0.16, *p* = 0.27; σ_Ε_: *r* = 0.39, *p* = 0.06; *N* = 18) or controls (μ: *r* = −0.34, *p* = 0.45; σ: *r* = −0.46, *p* = 0.41; σ_Ε_: *r* = −0.15, *p* = 0.29; *N* = 17). At a liberal threshold (uncorrected *p* < 0.05) there were positive correlations between σ and σ_Ε_ in controls (*r* = 0.52, *p* = 0.02; *N* = 17), and between μ and σ in patients in the ‘off’ condition (*r* = 0.49, *p* = 0.02; *N* = 18). No other significant correlations between the three LATER parameters were noted in patients or in controls.

There was a significant negative correlation between letter fluency and σ in the ‘on’ patient group (*r* = −0.54, *p* = 0.01, Bonferroni corrected for multiple comparisons; *N* = 18). At a more liberal threshold (uncorrected *p* < 0.05) there were positive correlations between animal fluency and μ in the ‘on’ patient group (*r* = 0.42, *p* = 0.05; *N* = 18) and between letter fluency and μ in the control group (*r* = 0.66, *p* = 0.02; *N* = 17). The other cognitive variables did not show any significant associations with any of the three LATER model parameters.

For control subjects, no difference is expected a priori between nominal ‘on’ and ‘off’ sessions. The correlations between sessions for the control group were highly significant for μ (*r* = 0.81, *p* < 0.001; *N* = 17), σ (*r* = 0.58, *p* < 0.01; *N* = 17), and σ_Ε_ (*r* = 0.66, *p* < 0.01; *N* = 17) indicating good replication of saccade parameters between sessions, within subjects.

### Voxel-based morphometry

**Fig. 2:**
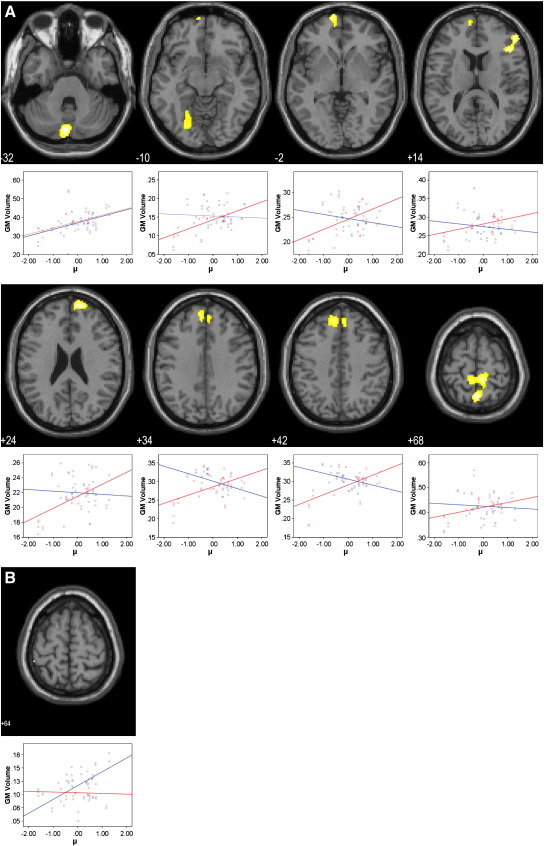
SPM{t} maps of voxelwise correlations between grey matter volume and the reciprocal saccade latency (μ) in patients (A) and controls (B). Anatomical localization as projected on axial sections of a normal MRI, spatially normalised into the MNI template (*p* < 0.05, FWE corrected for multiple comparisons) at the given *z* coordinates in MNI space. Sub-plots show mean-corrected μ vs. grey matter correlation data for controls (blue) and patients (red).

The voxel-based analyses revealed several clusters with a significant correlation between the three saccade latency parameters and grey matter volume both in the patient and in the control groups. In patients, the reciprocal latency (speed of response, μ) was positively correlated with grey matter volume in regions of the prefrontal cortex including the middle frontal gyrus and the polar prefrontal cortex together with the cerebellar vermis and the inferior semilunar lobule, the precuneus, the somatosensory association cortex and the fusiform gyrus (*p* < 0.05 FWE corrected; Fig. 2A and Table S1 in Supplement 1). The clusters in the superior frontal gyrus and the precuneus lay in regions which also had a trend towards grey matter reduction in patients compared with controls (*p* < 0.05 uncorrected). In the control group, there was a significant positive association between μ and grey matter volume in a single voxel of the inferior parietal lobule only (*p* < 0.05 FWE corrected; Fig. 2B and Table S1 in Supplement 1).

**Fig. 3:**
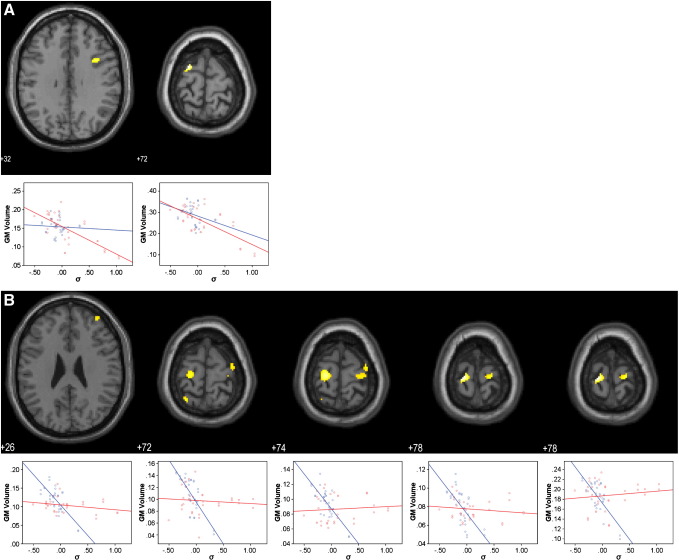
SPM{t} maps of voxelwise correlations between grey matter volume and the variability of the main saccade population (σ) in patients (A) and controls (B). Anatomical localization as projected on axial sections of a normal MRI, spatially normalised into the MNI template (*p* < 0.05, FWE corrected for multiple comparisons) at the given *z* coordinates in MNI space. Sub-plots show mean-corrected σ vs. grey matter correlation data for controls (blue) and patients (red).

σ was negatively associated with grey matter volume in the bilateral frontal eye fields in patients (*p* < 0.05 FWE corrected; Fig. 3A and Table S2 in Supplement 1). The right eye-field was additionally associated with a trend towards a grey matter difference between patients and controls (*p* < 0.05 uncorrected). In controls, there was also a strong negative correlation between σ and grey matter volume in the frontal eye fields bilaterally, and also the parietal eye fields, the premotor cortex, and the lateral prefrontal cortex (*p* < 0.05 FWE corrected; Fig. 3B and Table S2 in Supplement 1).

**Fig. 4:**
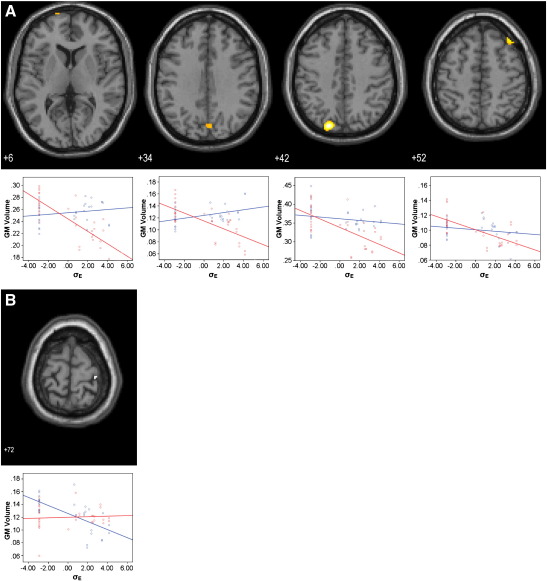
SPM{t} maps of voxelwise correlations between grey matter volume and the variability of the early saccade population (σ_E_) in patients (A) and controls (B). Anatomical localization as projected on axial sections of a normal MRI, spatially normalised into the MNI template (*p* < 0.05, FWE corrected for multiple comparisons) at the given *z* coordinates in MNI space. Sub-plots show group mean-corrected σ_E_ vs. grey matter correlation data for controls (blue) and patients (red).

In patients, σ_Ε_ correlated negatively with grey matter volume in the frontal eye fields, the precuneus, the middle frontal gyrus, and the prestriate cortex (*p* < 0.05 FWE corrected; Fig. 4A and Table S3 in Supplement 1). With the exception of the prestriate cortex, these associations were located in regions with a trend towards grey matter loss in patients versus controls (*p* < 0.05 uncorrected). In the control group, σ_Ε_ was negatively correlated with grey matter volume in the premotor cortex only (*p* < 0.05 FWE corrected; Fig. 4B and Table S3 in Supplement 1).

## Discussion

Saccadometry has potentially many applications in PD and associated disorders, including a role as a physiological biomarker for treatment monitoring and disease staging. Saccadic latency is of particular interest in this context because of its robust distributions, ease of measurement, reliability and associations with both cortical and sub-cortical functions. A few previous studies have reported evidence for latency changes in PD although no direct evidence existed for the neurobiological substrate of these saccadometric differences in PD.

The first finding of this study is that there were significant correlations between regional grey matter loss and each of the latency parameters from the LATER model in both patients and control subjects. These structure–function correlations included the frontal and parietal eye-fields and the cerebellum but also other association cortex associated with grey matter loss in PD. The second result is that latency was correlated with verbal fluency in patients with PD, but not motor severity (UPDRS III). Third, there were no latency differences between patients with PD and matched healthy control subjects, nor did we find any differences between patients ‘on’ or ‘off’ dopaminergic medication.

### Behavioural effects of Parkinson's disease

Using a similar paradigm to ours, Michell (Michell et al., 2006) also compared latency parameters between two groups of patients with PD and a healthy control group and reported significantly different reciprocal median latencies (speed of response, μ) for one of the two PD groups in the ‘on’ state. This difference was not observed in the ‘off’ state, although there was considerable inter-subject variability in PD and healthy ageing. In a second patient group, they also reported a significant influence of dopaminergic medication on the reciprocal latency (speed of response), with l-dopa prolonging latency particularly in a sub-set of 5/15 patients. The lack of effect of PD or dopaminergic medication in the current study could be a type II error, but our study had similar power to Michell et al. (2006) and similar saccadometry methods. One important difference is the patient profile. Our patients had longer disease duration, and in spite of similar severity when ‘on’ they were less severely ‘off’ after medication withdrawal and this may have been significant as might be the differences in dopaminergic drugs used between studies (van Eimeren et al., 2009).

The involvement of widespread cortical cerebral networks in saccadic control (Leigh and Kennard, 2004) and the impairment of certain aspects of saccadic eye movement in PD dementia and dementia with Lewy bodies (Mosimann et al., 2005) have raised the possibility of using saccadometry as a marker of cognitive dysfunction. In support of this, we found correlations between verbal fluency and saccadic parameters in patients and healthy control subjects. A better verbal fluency was associated with a shorter latency (higher μ) and lower promptness variability (σ) even in the absence of any significant correlations between motor function (UPDRS) and any of the three latency variables from the LATER model. In conjunction with previous reports, our findings suggest the value of saccadometry as a marker of cognitive function in PD and potentially other disorders associated with abnormalities of the prefrontal cortex (Garbutt et al., 2008).

### The brain structural substrate of saccadic latency

A striking finding of our study is the negative correlation between the variability of saccade latency (σ) and the grey matter volume of the frontal and parietal eye fields, regions associated with generation of saccades (Mort et al., 2003; Pierrot-Deseilligny et al., 1993; Rafal, 2006). The frontal eye field is located in the caudal part of the lateral prefrontal cortex and contributes to the transformation of visual signals into saccadic motor commands (Schall and Boucher, 2007). The activity of many neurons in this region increases steadily in the period leading up to a saccade, and as predicted by LATER, the rate of rise of their activity varies randomly from trial to trial (Hanes and Schall, 1996). The LATER model itself is agnostic as to the anatomy of saccade decisions, but is consonant with neurophysiological recordings of parietal and collicular neurons during saccade decisions.

The parietal eye field is thought to be specialised for the spatial processing essential to the planning of saccadic eye movements (Andersen et al., 1997). The importance of the parietal eye fields for triggering saccades is underlined by the effects of transcranial magnetic stimulation (TMS) on saccade latency. Vernet (Vernet et al., 2008) for example, showed that TMS over the posterior parietal cortex increased the saccadic latency in a group of young healthy volunteers. Therefore, grey matter volume decreases in the frontal and parietal eye fields are likely to affect the initiation of saccades in PD and healthy ageing, as we observed.

The frontal eye fields project via pontine nuclei to the cerebellar vermis (Van Opstal et al., 1996), the grey matter volume of which was positively correlated with the reciprocal latency (speed of response, μ) in our present study. In other words, higher grey matter volume in the vermis was associated with shorter latency. The dorsal vermis, together with the caudal fastigial nucleus, plays an important role in controlling the accuracy of saccades, encoding the time when a saccade has to stop to land on a target (Robinson and Fuchs, 2001).

Another cortical area important for saccadic control lies within the lateral prefrontal cortex; in the present study, higher grey matter volume of the lateral prefrontal cortex was associated with shorter latency and less variability of the saccades. The lateral prefrontal cortex is not only directly connected via the internal capsule with the superior colliculus (Gaymard et al., 2003), it also plays a central role for executive functions. Lesions in this area are associated with a number of executive deficits including unwanted gaze shifts (Pierrot-Deseilligny et al., 1991a). There are several possible explanations for the inhibitory role of the lateral prefrontal cortex. Neurons in the lateral prefrontal cortex may filter unwanted signals locally (Everling et al., 2002), have an inhibitory control over sensory areas (Kastner and Ungerleider, 2000), or suppress overt orienting of attention, i.e. attentional shifts with eye movements, toward irrelevant stimuli by direct inhibition of the superior colliculus (Pierrot-Deseilligny et al., 1991b). Therefore, the ability to suppress an erroneous, visually evoked saccade depends in part on the integrity of the lateral prefrontal cortex. The importance of executive control over saccades is also underlined by the correlation between verbal fluency and saccadic reciprocal latency and variability in the present study.

The heterogeneity of PD, and diversity of clinical groups in terms of stage and pathological distribution, introduces a high degree of non-random variance. This variance in structure and function reveals the covariance (or correlations) between GM volumes, saccades and cognition. It is not that structural integrity of prefrontal cortex is only important for saccades in patients, rather that healthy controls have greater integrity of prefrontal cortex as a feature of being healthy. However, not all GM atrophy in PD (or health) is relevant to saccades or cognition. By including healthy controls, and the groups and cognitive covariates in our models, we can identify and distinguish the functionally relevant changes in GM in the context of PD.

### Limitations of the study

The patients were recruited from a specialised university centre and may therefore not truly represent the whole population with PD. They were all moderately affected by the disease and on long term dopaminergic therapy. Therefore, the present findings might not apply for patients in more advanced or earlier PD stages. Furthermore, a modest number of patients were included and replication in larger samples would be helpful. Nonetheless, our VBM results were significant even after strict correction for multiple comparisons, giving good control of type I error. Finally, a larger cognitive test battery with more specific instruments would refine the association between cognitive function and saccade control.

## Conclusions and future perspectives

Few other human responses are as well understood as saccades, possessing dynamic properties that are easily measured and a well defined anatomical circuitry. Therefore, saccades have become a popular tool to measure oculomotor control and cognition. The deterioration of saccade control in neurodegenerative disorders has led some authors to suggest that saccadometry may be a reliable and easy to use biomarker.

Our present study adds several important pieces of evidence that qualify this suggestion. Both our behavioural and imaging findings point to an association between saccade latency, executive function and the prefrontal cortex. Our findings also suggest that saccadic changes are associated with PD-related atrophy within a well defined oculomotor network that includes the frontal and parietal eye fields and the cerebellar vermis. Therefore, in PD, saccadometry may provide objective important information on the severity of cognitive dysfunction and at the same time offer a rapid and non-invasive correlate of focal brain atrophy. However, our results do not support the use of saccadometry as a diagnostic biomarker to distinguish PD from normal ageing.

## Figures and Tables

**Table 1:** Study sample characteristics.

Characteristic	Patients (*N* = 18)	Controls (*N* = 17)	*p*
Age, years	65.33 (7.77)	67.00 (6.18)	0.49
Men:women	10:8	8:9	0.62
Duration of disease, years	7.56 (3.09)	NA	NA
MMSE	28.18 (1.67)	29.14 (0.69)	0.97
Verbal fluency, animals/1 min	20.88 (6.17)	23.63 (4.24)	0.27
Verbal fluency, p-words/1 min	16.35 (6.56)	16.00 (4.76)	0.88
UPDRS-III off	30.72 (9.09)	NA	NA
UPDRS-III on	19.28 (8.40)	NA	NA
Levodopa equivalent dose, mg	630.56 (335.25)	NA	NA
Brain volume, ml	1926.83 (503.97)	2038.33 (425.21)	0.49

Mean values (standard deviation) where appropriate, NA: not applicable. Probability values based on unpaired *t*-tests or chi-squared distributions as appropriate.

**Table 2:** Saccade parameters patients vs. controls.

Parameter	Patients (*N* = 18)	Controls (*N* = 17)	*p*
μ off	4.71 (1.12)	4.77 (0.70)	0.85
μ on	4.57 (0.86)	4.77 (0.60)	0.44
σ off	1.38 (0.63)	1.12 (0.21)	0.11
σ on	1.21 (0.41)	1.05 (0.15)	0.14
σ_Ε_ off	3.54 (2.40)	2.45 (2.71)	0.23
σ_Ε_ on	2.76 (2.85)	3.03 (2.76)	0.78

Mean values (standard deviation) where appropriate, NA: not applicable.

There were also no significant differences between patients ‘on’ and ‘off’ (see Results section).
