# Supplementary material for: Saccadic latency in Parkinson's disease correlates with executive function and brain atrophy, but not motor severity
Source: Neurobiol Dis. 2011 Jul;43(1):79–85. doi: 10.1016/j.nbd.2011.01.032 (PMC3102178; doi:10.1016/j.nbd.2011.01.032)

**Supplement 1**

**Methods**

**Recruitment of patients and control subjects**

The diagnosis of idiopathic PD was established by an experienced neurologist according to UK PD Society Brain Bank clinical diagnostic criteria. Patients were excluded if they suffered from major depression or dementia, had a contraindication for MRI, were currently not on dopaminergic medication, or had previously suffered adverse reactions to withdrawal or delay of dopaminergic medication.

Participants were assessed on two sessions, at approximately the same time of day and at least one week apart. They were asked to take their usual medication on one day to be in a dopaminergic 'on' state. On the other day they were asked to stop their medication at least 12 hours before the scan in the case of short acting dopaminergic preparations (or at least 24 h in the case of long acting preparations) and were therefore in a relative 'off' state. Although both the 'on' and the 'off' conditions are only relative, the withdrawal period of dopaminergic medication was sufficient to cause a clinically significant 'off' state and a significant increase in the UPDRS III score. Although not treated with levodopa, controls were also scanned twice and randomly assigned to a nominal 'on' or 'off' session to balance for session effects including practice. The order of testing in either ‘on’ or ’off’ days was randomly permuted within each group.

The neuropsychological assessment was conducted after completion of the 'on' scanning session. It included the Mini-Mental-State Examination (MMSE), and tests of animal fluency (animals/1 min) and letter fluency (words beginning with the letter p/1 min). The severity of PD motor symptoms was assessed by the UPDRS III immediately before scanning. Saccadometry was performed on both the 'on' and 'off' days. Information on age, gender, duration of disease, and current medication was available on all cases. Doses of dopaminergic medication at the time of assessment were recorded and converted to equivalent levodopa doses using an established formula (equivalent levodopa dose = [levodopa (x 1.2 if COMT inhibitor) (x 1.2 if 10 mg selegiline OR 1.1 if 5mg selegiline)] + [pramipexole x 400] + [ropinirole x 40] + [cabergoline x 160] + [pergolide x 200] + [bromocriptine x10] + [lisuride x 160]; all doses in mg).

**Acquisition of the saccadometry data**

The small lightweight saccadometer is mounted on the forehead and rests on the bridge of the nose, projecting visual targets with three red lasers (a central fixation target and two peripheral targets 10° horizontally on each side). The participants sat 1.5m from a matt white wall, on which the targets were projected; because the targets move with the head, no head restraint is needed.

Automated calibration trials were performed before the actual experiment. A saccadic step task paradigm was then implemented automatically. The fixation target was illuminated for a random period of 1 - 2 s, after which one of the two peripheral targets was illuminated with equal probability. The participants were instructed to look at the targets and follow their movement. After the saccade to the peripheral target, the target remained illuminated for 200 ms before the target returned to the central position and the next trial was initiated. A complete session typically consisted of 300 such trials with a break after 150 trials or upon request.

Saccadometry data was transferred to a standard personal computer and a pre-processing was performed in the LatencyMeter software v4.7 (ober-consulting.com). This software eliminates invalid trials (> 200 valid trials were available for all participants) and calculates latencies based on instantaneous velocity and acceleration. The data was subsequently transferred to SPIC software (cudos.ac.uk/spic.html) for latency distribution analyses including the estimation of the best-fit LATER parameters by minimisation of the Kolmogorov-Smirnov statistic. LATER parameters of the 'on' sessions were used for the MRI analyses as no behavioural differences were found between ‘on’ and ‘off’ sessions. Left and right saccades were combined.

**Acquisition and pre-processing of the structural MRI data**

Structural MRI scans were obtained on a Siemens Tim Trio 3T scanner running a high resolution 3D magnetization prepared rapid gradient echo (MPRAGE) sequence (TR 2250 ms, TE 2.99 ms, FA 9 degrees, IT 900 ms, 256 x 256 x 192 isotropic 1 mm voxels). Image pre-processing was performed in SPM5 (Wellcome Functional Imaging Laboratory, London, UK; fil.ion.ucl.ac.uk/spm) implemented in Matlab v7 (The Mathworks Inc., Natick, MA, USA) running on a standard personal computer.

Data underwent an iterative unified segmentation and normalisation procedure into three tissue compartments (grey matter; white matter; cerebrospinal fluid) with bias correction and warping to the Montreal Neurological Institute (MNI) T1 template. The grey matter images were modulated due to the focus of the present study on grey matter volume rather than grey matter density, and then smoothed by an isotropic Gaussian kernel with 12 mm FWHM. Voxels with a grey matter value greater than 0.1 (maximum value, 1) were included in the subsequent analysis.

Visual rating of the individual MRI-scans was performed by experienced clinical readers to screen for structural abnormalities, tumors or signs of major vascular pathology. It was verified that MRI-data were compatible with the original clinical diagnosis on the basis of all acquired MRI-sequences.

**Data analysis**

Voxel-based morphometry (VBM) in SPM5 software used general linear models to estimate the associations between the three LATER parameters and grey matter volume in patients and controls. Each model included regressors that separately specified each control and patient group acquired on different sessions ('on' and 'off'), one LATER parameter, and total grey matter volume as a covariate. Total grey matter volume was calculated by summing the product of grey matter volume per voxel over all voxels. Further covariates were not included; previously published literature carrying out VBM have chosen from a wide range of nuisance covariates including: grey matter; total intracranial volume (TIV); TIV and age; TIV, age and gender; or the sum of grey and white matter, i.e. total brain volume. Age and gender covary with grey matter volume or TIV, reducing the value of including all three as covariates. The correct choice of covariate depends in part on the nature of the inference required – for example, that local grey matter atrophy is disproportionate to subject specific differences in total grey matter. The inclusion of the individual levodopa equivalent doses and the verbal fluency test results as further covariates in separate analyses did not significantly alter the results (not shown). Scans from both sessions were included and all covariates were mean corrected within group.

Clinical and demographic variables were analyzed in the Predictive Analytics Software package (PASW) v17 (The SPSS Inc., Chicago, IL, USA). Mean values were examined for significant differences using Student's unpaired t-test to compare groups and paired t-tests for comparing the patients ‘on’ vs. ‘off’. Absolute frequencies were compared using chi-square tests. Associations between clinical test results and LATER parameters were explored using non-parametric, one-tailed tests. In detail, associations between the LATER parameters μ, σ, and σ_E_ and the variables UPDRS III (‘on’ and ‘off’), MMSE, animal and letter fluency, and whole brain volume were tested. Results with a p < 0.05 were regarded significant. A Bonferroni correction for multiple comparisons was applied within each set of correlations for the three LATER parameters.

**Table S1.** Peak correlations between grey matter volume and the median reciprocal saccade latency (μ)

| Patients | | | | | | Controls | | | | | |
| --- | --- | --- | --- | --- | --- | --- | --- | --- | --- | --- | --- |
| Region | x | y | z | Z-score | cluster | Region | x | y | Z | Z-score | cluster |
| **lUV** | **-4** | **-80** | **-32** | **6.09** | **494** | **lIPL** | **-46** | **-38** | **64** | **4.61** | **1** |
| lISS | -8 | -74 | -48 | 5.02 |  |  |  |  |  |  |  |
| **rPC** | **2** | **-60** | **68** | **5.88** | **976** |  |  |  |  |  |  |
| rPCG | 8 | -36 | 70 | 5.82 |  |  |  |  |  |  |  |
| lPCG | 0 | -36 | 78 | 5.71 |  |  |  |  |  |  |  |
| **rSFG** | **14** | **62** | **24** | **5.87** | **233** |  |  |  |  |  |  |
| **rMFG** | **46** | **26** | **14** | **5.81** | **220** |  |  |  |  |  |  |
| rMFG | 52 | 40 | 12 | 5.78 |  |  |  |  |  |  |  |
| **lMeFG** | **-10** | **68** | **-2** | **5.68** | **262** |  |  |  |  |  |  |
| lMeFG | -8 | 58 | 4 | 5.25 |  |  |  |  |  |  |  |
| **lSFG** | **-6** | **40** | **42** | **5.47** | **309** |  |  |  |  |  |  |
| lMeFG | -6 | 50 | 34 | 5.27 |  |  |  |  |  |  |  |
| **rMeFG** | **6** | **40** | **38** | **5.43** | **122** |  |  |  |  |  |  |
| **lDC** | **-22** | **-74** | **-10** | **5.15** | **181** |  |  |  |  |  |  |
| lFFG | -24 | -58 | -8 | 4.96 |  |  |  |  |  |  |  |

Significant brain clusters were labelled in Talairach daemon software (talairach.org/daemon.html) after conversion of MNI (bic.mni.mcgill.ca) to Talairach coordinates (57) in GingerAle software (brainmap.org/ale).

Bold markings delineate a cluster, subsequent non-bold markings identify further peaks within the same cluster; brain regions are indicated by MNI coordinates; cluster: extent of contiguous voxels within the cluster; r: right, l: left; UV: uvula; ISS: inferior semilunar lobule; PC: precuneus; PCG: postcentral gyrus; SFG: superior frontal gyrus; MFG: middle frontal gyrus; MeFG: medial frontal gyrus; DC: declive; FFG: fusiform gyrus; IPL: inferior parietal lobule

**Table S2.** Peak correlations between grey matter volume and the variability of the main population of saccade latency (σ)

| Patients | | | | | | Controls | | | | | |
| --- | --- | --- | --- | --- | --- | --- | --- | --- | --- | --- | --- |
| Region | x | y | z | Z-score | cluster | Region | x | y | Z | Z-score | cluster |
| **lSFG** | **-22** | **2** | **72** | **5.92** | **62** | **lPCG** | **-18** | **-26** | **78** | **6.13** | **136** |
| rPCG | 36 | 10 | 32 | 5.83 |  | **rPrCG** | **20** | **-24** | **78** |  | **58** |
|  |  |  |  |  |  | **lSPL** | **-26** | **-56** | **72** |  | **19** |
|  |  |  |  |  |  | **rPrCG** | **36** | **-12** | **74** |  | **23** |
|  |  |  |  |  |  | **rMFG** | **38** | **52** | **26** |  | **22** |

SFG: superior frontal gyrus; PCG: precentral gyrus; PrCG: precentral gyrus; SPL: superior parietal lobule; MFG: middle frontal gyrus

**Table S3.** Peak correlations between grey matter volume and the variability of the early population of saccade latency (σ_E_)

| Patients | | | | | | Controls | | | | | |
| --- | --- | --- | --- | --- | --- | --- | --- | --- | --- | --- | --- |
| Region | x | y | z | Z-score | cluster | Region | x | y | Z | Z-score | cluster |
| **lPC** | **-18** | **-82** | **42** | **8.99** | **181** | **rPCG** | **34** | **-26** | **72** | **4.64** | **5** |
| **rSFG** | **40** | **30** | **52** | **6.57** | **58** |  |  |  |  |  |  |
| **rCN** | **4** | **-76** | **34** | **5.43** | **30** |  |  |  |  |  |  |
| **lSFG** | **-16** | **72** | **6** | **4.78** | **6** |  |  |  |  |  |  |

PC: precuneus; SFG: superior frontal gyrus; CN: cuneus; PCG: precentral gyrus

**Figure S1.** An individual patient’s reciprobit plot. Cumulative frequency is plotted on a probability scale as a function of reciprocal latency. Processes obeying LATER will then generate a single straight line; however, in certain situations there is a separate population of early latency saccades that fall on a subsidiary line with shallower slope.


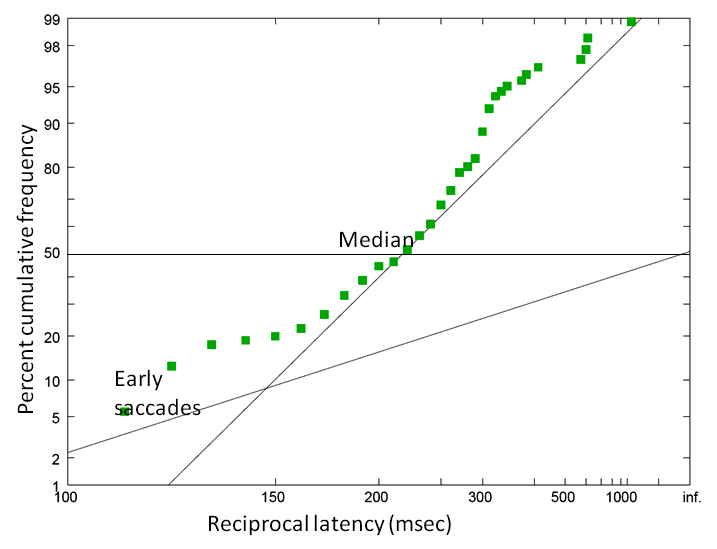

Supplement: Supplementary file 1 — Supplementary materials [file mmc1.doc]
